# Extracellular Vesicles and Alveolar Epithelial-Capillary Barrier Disruption in Acute Respiratory Distress Syndrome: Pathophysiological Role and Therapeutic Potential

**DOI:** 10.3389/fphys.2021.752287

**Published:** 2021-11-23

**Authors:** Sergio Esquivel-Ruiz, Paloma González-Rodríguez, José A. Lorente, Francisco Pérez-Vizcaíno, Raquel Herrero, Laura Moreno

**Affiliations:** ^1^Department of Pharmacology and Toxicology, School of Medicine, University Complutense of Madrid, Instituto de Investigación Sanitaria Gregorio Marañón (IiSGM), Madrid, Spain; ^2^Ciber de Enfermedades Respiratorias (CIBERES), Madrid, Spain; ^3^Department of Critical Care, Hospital Universitario de Getafe, Madrid, Spain; ^4^Clinical Section, School of Medicine, European University of Madrid, Madrid, Spain

**Keywords:** barrier dysfunction, airway epithelium, pulmonary endothelium, mesenchymal stem cells, extracellular vesicles (EVs), preconditioning

## Abstract

Extracellular vesicles (EVs) mediate intercellular communication by transferring genetic material, proteins and organelles between different cells types in both health and disease. Recent evidence suggests that these vesicles, more than simply diagnostic markers, are key mediators of the pathophysiology of acute respiratory distress syndrome (ARDS) and other lung diseases. In this review, we will discuss the contribution of EVs released by pulmonary structural cells (alveolar epithelial and endothelial cells) and immune cells in these diseases, with particular attention to their ability to modulate inflammation and alveolar-capillary barrier disruption, a hallmark of ARDS. EVs also offer a unique opportunity to develop new therapeutics for the treatment of ARDS. Evidences supporting the ability of stem cell-derived EVs to attenuate the lung injury and ongoing strategies to improve their therapeutic potential are also discussed.

## Introduction

Acute respiratory distress syndrome (ARDS) is the major cause of acute respiratory failure in the intensive care units (ICUs) and carries a high mortality rate (Thompson et al., 2017). Risk factors for this condition include infection, trauma or other systemic conditions. Management is exclusively supportive and the lack of approved pharmacological therapies reflects major deficiencies in our understanding of the pathogenesis of ARDS. Although studies defining appropriate ventilator management have improved patient outcomes, the incidence and mortality associated with ARDS remains unacceptably high. In late December 2019, an outbreak of a novel severe acute respiratory syndrome coronavirus 2 (SARS-CoV-2) was first identified. Its manifestations can range from asymptomatic or mild respiratory infection to severe ARDS and death. With a high rate of transmission and mortality, the SARS-CoV-2 infection (COVID-19) has become a global threat which demands effective treatments beyond supportive therapies (Zheng, 2020).

The pathological hallmark of ARDS is the diffuse alveolar damage with an early alveolar epithelial-capillary barrier disruption. ARDS is characterized by pulmonary edema and alveolar collapse accompanied by ventilation-perfusion mismatch and severe arterial hypoxemia (Thompson et al., 2017). In these patients, the injured alveoli are characterized by the presence of an intense inflammatory response with leukocyte infiltration, activation of pro-coagulant processes in the alveolar air spaces and in the microvasculature, and damage of epithelial and endothelial cells. All these events contribute to the breakdown of the alveolar-epithelial barrier and, consequently, to the formation of alveolar protein-rich edema. Such pulmonary edema is a major factor for hypoxemia and one of the earliest events in ARDS. Once ARDS develops, patients may present pulmonary vascular dysfunction and pulmonary hypertension which is independently associated with poor outcomes in patients with ARDS (Bull et al., 2010). Uncontrolled inflammation can also lead to the massive release of inflammatory mediators (such as interleukin [IL]-1β, IL-6, IL-8 or tumor necrosis factor α [TNF-α]), causing the so-called “cytokine storm” which results in vascular inflammation, thrombosis and vasodilation and may lead to multiorgan dysfunction (Thompson et al., 2017; Zheng, 2020). Besides the formation of thrombi in the pulmonary microvasculature, disseminated intravascular coagulation (DIC) is also a frequent complication in patients with severe ARDS that can be clinically expressed by excessive hemorrhage and ischemic necrosis of extremities. In addition, the onset of DIC has been associated with deterioration of lung functions with worsening hypoxemia and a progressive fall in pulmonary compliance (Gando et al., 2004). Platelet activation and pulmonary-capillary endothelial-platelet interaction may be one of the major and earliest mechanisms involved in both local lung and systemic coagulation in these patients. Furthermore, increasing evidence shows the relevant role of activated platelets and their vasoactive and procoagulant products in the increase of pulmonary capillary permeability that contributes to lung edema formation in ARDS (Zarbock et al., 2006).

## Structure and Function of Alveolar Epithelial-Capillary Barrier

In the lung, the alveolar-capillary membrane separates the alveolar airspace from the capillary lumen and comprises a complex architecture optimized to exert its multiple functions that include gas exchange (oxygen is diffused into the capillaries and carbon dioxide released from the capillaries into the air space). The alveolar-capillary membrane has several layers: a lining fluid layer containing the surfactant, the epithelial barrier and its basement membrane, a thin interstitial space with a biologically active extracellular matrix (ECM), a capillary basement membrane and the capillary endothelium (Weibel, 1973; Maina and West, 2005; Knudsen and Ochs, 2018). Between these epithelial and endothelial layers there are also resident and migratory leukocytes, as well as a population of mesenchymal stromal cells, such as pericytes and resident fibroblasts (Barron et al., 2016).

The alveolar epithelium is a tight barrier that restricts the passage of water, electrolytes, and small hydrophilic solutes from the insterstitium to the air spaces, allowing at the same time the diffusion of carbon dioxide and oxygen (Taylor and Gaar, 1970; Weibel, 2015). The alveolar epithelium is composed by flat alveolar type I (ATI) cells and cuboidal shaped alveolar type II (ATII) cells. The ATII cells secrete surfactant, the critical factor that reduces surface tension, enabling the alveoli to remain open and facilitating gas exchange. ATII also can differentiate to replace damaged type I cells (Matthay et al., 1993; Mason and Dobbs, 2016). Both ATI and ATII cells have the capacity to absorb excess fluid from the airspaces to the interstitium by vectorial ion transport, primarily promoted by epithelial sodium channels (ENaC) and basolateral Na^+^/K^+^-ATPase pumps (Matthay, 2014; Matthay et al., 2019). Thus, proper function of these channels is essential for the reabsorption of edema and are key elements for a clear improvement of the disease in patients with sepsis and ARDS (Ware and Matthay, 2001; Zeyed et al., 2012). However, this capability of alveolar fluid clearance not only depends on intact ion transport channels in the epithelial cells, but also on the integrity of the junctions between ATI and ATII cells. Once into the insterstitium, the edema fluid is removed to systemic circulation by the lymphatics. Although more permeable than the epithelium, the capillary endothelium in the alveoli forms a semipermeable barrier that limits the extravasation of plasma and its macromolecules from the vascular lumen to the instertitium. Both epithelial and endothelial barrier functions and permeability are governed by intercellular junctions (Herrero et al., 2018). These intercellular junctions between neighboring cells in the epithelium and endothelium are mainly formed by apical tight junctions (TJs) and the underlying adherens junctions (AJ), and linked to the cellular cytoskeleton via numerous adaptor proteins. AJs are composed of cadherins, mainly vascular endothelial cadherin (VE-cadherin) that regulate the paracellular transport between blood and interstitium, consequently determine leukocyte migration and edema formation during ARDS (Millar et al., 2016).

In general, TJs control paracellular transport, maintain cellular polarity, establish separate intercellular compartments, regulate a variety of intracellular signals, and control the transcellular transport. Occludins, claudins, and zonula occludens (ZO) are essential components of tight junctions in the alveolar epithelium that constitute the main structure to regulate the passage of water and solute from the interstitial to the alveolar space, and to prevent the passage of pathogens and toxins from the air space into the systemic circulation (Zemans and Matthay, 2004; Yanagi et al., 2015; Herrero et al., 2018). Thus, alteration of the epithelial TJs results in protein-rich edema formation, and passage of infectious agents, exogenous toxins and endogenous products into the systemic circulation, exposing other organs and contributing to multiorgan failure (Denker and Nigam, 1998; Schneeberger and Lynch, 2004; Herrero et al., 2018). Endothelial junctional proteins also play important roles in tissue integrity as well as in vascular permeability, leukocyte extravasation, and angiogenesis (Wallez and Huber, 2008). Specifically, intercellular cell adhesion molecule- 1 (ICAM-1) and vascular cell adhesion molecule-1 (VCAM-1) control the adhesion of leukocytes to endothelial cells and facilitate their subsequent transendothelial migration via the platelet-endothelial cell adhesion molecule-1 (PECAM-1), contributing to the inflammatory process (Millar et al., 2016; Villar et al., 2019).

The ECM, composed of a highly dynamic complex of fibrous proteins, glycoproteins, and proteoglycans, is crucial to maintain the epithelial and endothelial barrier function by regulating cell-cell interactions. ECM also modulates cell survival, proliferation, migration and differentiation, and play an important role in tissue repair. Resident fibroblasts in the interstitium are mainly responsible for ECM production (White, 2015; Barron et al., 2016). Changes in the composition and mechanic properties of the ECM have been shown to modify the expression of TJs and the barrier function in alveolar epithelial and endothelial cells, contributing to lung edema formation (Rocco et al., 2001; Pelosi et al., 2007; Koval et al., 2010; Mammoto et al., 2013). Post-translational modifications (e.g., enzymatic and chemical crosslinking, transglutamination, glycation and glycosylation, oxidation, and citrullination), are known to affect the structural and/or functional diversity of ECM proteins. The structural organization and degradation of the ECM are controlled by the proteolytic action of many proteases, including the superfamily of metalloproteinases that in turn comprises adamalysins (e.g., ADAMS, ADAMTS), and matrix metalloproteinases (MMPs) and their inhibitors (the tissue inhibitors of metalloproteinases-TIMPs). These enzymes are expressed by inflammatory cells (mainly macrophages) and stromal cells (including fibroblasts, endothelial and epithelial cells). In this line, a growing body of evidence shows a role of MMP-1, -2, -3, -7, -8, and -9, expressed by inflammatory, mesenchymal and epithelial cells, in the development and repair of the alveolar-capillary damage in ARDS (Davey et al., 2011). The oxidation of ECM proteins by reactive oxygen species (ROS) further modulates characteristics of the ECM. It is well known that increased levels of ROS are present in the alveoli of patients with ARDS that may alter ECM properties, having an impact on cell-ECM interaction and alveolar permeability (Watson et al., 2016).

In this interstitial space the pericytes and resident fibroblasts, not only play a relevant role in the maintenance of the normal vascular and epithelial functions in the lung, but also contribute to lung injury in pathological conditions. Pericytes underlie and envelop capillaries, forming intimate contacts with adjacent endothelial cells that control vessel integrity, angiogenesis and capillary permeability. Resident fibroblasts are mainly located beneath epithelial cells or are scattered through the interstitium. These mesenchymal stromal cells generate and remodel extracellular matrix, regulate the vasculature, help maintain and restore the epithelial barrier structure and function, and control immune cell activity and migration. These cells are the main source of myofibroblasts in the lung during development and after injury, contributing in the latter to lung fibrosis (Rock et al., 2011; Hung et al., 2013; Barron et al., 2016).

The lung has two main types of macrophages that reside in different anatomical compartments, namely interstitial and alveolar macrophages. Whereas alveolar macrophages (AM) are predominantly of embryonic origin, interstitial macrophages (IM) are mainly derived by blood monocytes. Both types of macrophages have an important role in host-defense as part of the local innate immune response in the lung (Evren et al., 2020). AM are in close communication with the alveolar epithelial cells (AECs) by different mechanisms including mediators, membrane glycoproteins and their receptors, gap junction channels, and extracellular vesicles (EVs). Thanks to this communication, AM regulate the alveolar epithelial barrier function and permeability, while exerting other functions such as barrier immunity, surfactant clearance, and removal of foreign particles. In pathological conditions, activated AM cause epithelial cells to produce anti-microbial peptides and a variety of mediators that not only modulate immune responses, but also contribute to epithelial barrier dysfunction via alteration of the TJ proteins (Bissonnette et al., 2020). Interstitial macrophages, located in the space between the lung epithelium and capillaries in the airway, perform antigen presentation and contribute to tissue remodeling in the lung (Koch et al., 2017). In contrast to AM, the location of IM in the alveoli and their contribution to ARDS have not been totally elucidated.

Multiple clinical and experimental studies have provided relevant information about the different mechanisms involved in acute lung injury (ALI) and recovery. One of the main conclusions is that recovery from lung injury requires the repair of the endothelial barrier as well as the reestablishment of the normal functions of the alveolar epithelial barrier capable of secreting surfactant, preventing the passage of infectious/toxics agents into systemic circulation, and removing alveolar edema (Herrero et al., 2018; Matthay et al., 2019). Increasing studies are focusing on EVs released by pulmonary structural cells, immune cells and mesenchymal stem cells and their potential contribution to the development or resolution of lung injury.

## Extracellular Vesicles: Biogenesis and Function

Extracellular vesicles represent a heterogeneous range of membrane enclosed spheres of varying size that are secreted by a variety of cell types, including T cells, B cells, dendritic cells, platelets, mast cells, epithelial cells, endothelial cells, neuronal cells, cancerous cells, oligodendrocytes, Schwann cells, embryonic cells, and mesenchymal stem cells (MSCs) (Raposo and Stoorvogel, 2013; Borgovan et al., 2019). They are shed or secreted from these cell types under various physiologic and pathologic conditions into the circulation or surrounding body fluids, including blood or bronchoalveolar lavage fluid (BALF) (Witwer et al., 2013; Cocucci and Meldolesi, 2015; Konala et al., 2016; Borgovan et al., 2019).

The International Society of Extracellular Vesicles (ISEV) establishes the minimum requirements for the collection and pre-processing of samples and for the separation, concentration, and characterization of EVs, as well as the steps to demonstrate that a function is associated specifically with EVs (Thery et al., 2018; Nieuwland et al., 2020). According to the ISEV, three main sub-groups of EVs have been classified based on their size, membrane composition and biogenesis, as shown in Table 1 (Raposo and Stoorvogel, 2013; Witwer et al., 2013). Apoptotic bodies (50–5000 nm) are the largest EVs and are formed during cellular apoptosis by cell membrane-blebbing. Apoptotic bodies contain histones and genomic DNA. Microvesicles (MVs; 100–1000 nm) are shed via the outward blebbing of the plasma membrane, allowing retention of the membrane proteins of the parent cell. MVs are rich in the surface marker CD40, integrins and selectins as well as cholesterol, sphingomyelin, and ceramide. Exosomes (40–120 nm) are the smallest subgroup and are released after multiple vesicular bodies fuse with the plasma membrane. Exosomes may express distinct biomarkers, including tetraspanins (CD61, CD63, or CD81), ESCRT proteins (TSG101 and Alix), flotillin, and heat shock proteins, as well as high acetylcholinesterase activity (Crescitelli et al., 2013; Raposo and Stoorvogel, 2013; Cocucci and Meldolesi, 2015; Yanez-Mo et al., 2015; Borgovan et al., 2019).

**TABLE 1:** Types of extracellular vesicles based on their size and biogenesis pathways.

Characteristic	Exosomes	Microvesicles	Apoptotic bodies
Size	40–120 nm	100–1000 nm	50–5000 nm
Morphology	Cup-shaped	Heterogeneous	Heterogeneous
Formation mechanism	Multivesicular body	Plasma membrane	Plasma membrane
Pathways	(1) ESCRT-dependent (2) Tetraspanin-dependent (3) Ceramide-dependent	(1) Ca^+2^-dependent (2) Stimuli-dependent	Apoptosis-related pathways
Content	Proteins, lipids, and nucleic acids	Proteins, lipids, and nucleic acids	Nuclear fractions, cell organelles
Markers	Alix, Tsg101, tetraspanins (CD81, CD63, CD9), flotillin, heat shock proteins. High acetylcholinesterase activity	Integrins, selectins, CD40	Annexin V, phosphatidylserine
	Definitive markers for the different EV subpopulations do not exist.		

*CD, cluster of differentiation; ESCRT, endosomal sorting complexes required for transport; EV, extracellular vesicle; TSG101, tumor susceptibility gene 101.*

Extracellular vesicles are carriers of biologically active molecules (nucleic acids, proteins and lipids), whose composition vary based on the parent cell phenotype and biological state (Keerthikumar et al., 2016; Pathan et al., 2019; Xie et al., 2020). Proteins carried by EVs include chemokines, and inflammatory cytokines, integrins, growth factors, enzymes or even cytoskeletal components (Gutierrez-Vazquez et al., 2013; Keerthikumar et al., 2016; Maas et al., 2017; Mardpour et al., 2019). Nucleic acid cargo in EVs comprise mitochondrial and genomic DNA, small non-coding RNA species (such as microRNA or tRNA, small nucleolar RNA, and small nuclear RNA) and long non-coding RNA species (Kalra et al., 2012; Keerthikumar et al., 2016; Xie et al., 2020). EVs are also an important source of lipids, including sphingomyelin, ceramides, phosphatidylserine (PS), cholesterol or saturated fatty acids (Gutierrez-Vazquez et al., 2013; Chatterjee et al., 2020b).

Increasing evidences suggest that EVs mediate intercellular communication by transferring their cargo to recipient cells and are able to modulate physiological and pathophysiological process in both parent and recipient cells, including the induction and the resolution of lung injury and inflammation (Lee et al., 2018a; Lanyu and Feilong, 2019). In this review, we will discuss the potential pathophysiological and therapeutic role of EVs in ARDS. Since exosomes and MVs can overlap in the size range and current methods are unable to separate these populations efficiently, the ISEV has encouraged using the generic term of “extracellular vesicles” (Thery et al., 2018). Following these recommendations, we will use the term EVs, without making distinctions between exosomes or MVs, since the vast majority of studies have not been able to determine the specific biogenesis pathway for a given vesicle.

## Contribution of Extracellular Vesicles to the Pathogenesis of Acute Respiratory Distress Syndrome: Focus on Alveolar Epithelial-Capillary Barrier Disruption

Increased levels of EVs have been detected in BALF following either pulmonary viral and bacterial infections (Mills et al., 2019) or injury (hyperoxia, ventilator-induced lung injury-VILI, acid installation) (Lanyu and Feilong, 2019; McVey et al., 2019). Interestingly, a study carried out in mice revealed that BALF-EVs from sterile stimuli (hyperoxia-induced ALI) were mainly derived from ATI epithelial cells (enriched in cytokeratin and podoplanin), whereas BALF-EVs from infection-induced injury were mainly released by AM (enriched in CD68). This study showed that these two types of EVs activated AM through Toll-like receptor (TLR) pathways, although differential TLR pathways and signaling cascades were involved. In this regard, epithelial cell-derived EVs (non-infectious stimuli-induced EVs) activated TLR2, Myd88, TNF-α, and IL-6 expression in recipient macrophages, whereas macrophage-derived EVs (infectious stimuli-induced EVs) upregulated TLR6, TLR9, CD80, IL-1β, and IL-10 in recipient macrophages (Lee et al., 2018b). Both TLR2 and TLR6 are key receptors in the nuclear factor kappa B (NFκB)-mediated inflammation in many lung diseases, including ARDS (Lafferty et al., 2010).

A growing body of evidence shows that BALF-EVs significantly contribute to the development of lung inflammation and the progression of pulmonary damage during ARDS (Lee et al., 2018b). In patients with ARDS, secretory phospholipase A2 (sPLA2) is increased in BALF-EVs at an early state compared to patients without ARDS (Papadopoulos et al., 2020). sPLA2 is an oxidative stress-induced inflammation factor that activates inflammation and hydrolyzes lung surfactant phospholipids, contributing to lung collapse (Kim et al., 1995). In mouse models of ventilator-induced lung injury (VILI), BALF-EVs are enriched in IL-1β, IL-6, and TNF-α compared to non-ventilated control animals (Dai et al., 2019). Certain miRNAs were also detected in BALF-EVs of lipopolysaccharide (LPS)-induced ARDS in animals, specifically miR-466g, miR-466m-5p, miR-155, and miR-146a (Shikano et al., 2019). BALF-EVs enriched with miR-466g and miR-466m-5p activated the nod-like receptor family pyrin domain containing 3 (NLRP3) inflammasome in macrophages culture, inducing the IL-1 production by macrophages (Shikano et al., 2019). Cultured AECs treated with BALF-EVs enriched in miR-155 and miR-146a overexpress pro-inflammatory mediators, such as TNF-α and IL-6, and downregulate the expression of zonula occludens 1 (ZO-1) (Yuan et al., 2018), suggesting their role in epithelial barrier disruption.

Circulating EVs are also increased in ARDS models, including infection-related, endotoxin-induced and sterile lung injury (Letsiou et al., 2015; Moon et al., 2015; Pan et al., 2017; Jiang et al., 2019; Chatterjee et al., 2020a). Pneumonia induced by *Escherichia coli* in an *ex vivo* model of perfused human lung causes the release of EVs, enriched in TNF-α and IL-6, into the perfusate. Interestingly, these pneumonia-induced EVs, mainly derived from endothelial cells and platelets, are capable to cause lung damage when administered to healthy lung (Liu et al., 2019). Indeed, increased levels of endothelial-derived EVs have been associated with endothelial dysfunction (Letsiou and Bauer, 2018), gas-exchange deterioration (Cabrera-Benitez et al., 2015; Pan et al., 2017) and higher mortality (Sun et al., 2012) in ARDS patients. Also, it is crucial to determine the cellular origin of circulating EVs, since a protective role for some EVs has also been identified. For example, Shaver et al. (2017) found that elevated concentrations of EVs in plasma of patients admitted to ICU were associated with a reduced risk of developing ARDS, whereas increased levels of leukocyte-derived EVs in BALF, during the early stages of the disease, were associated with increased survival in ARDS patients (Guervilly et al., 2011).

Extracellular vesicles can also serve as vehicles of mature infectious virus particles between cells, which can increase the infectivity to host cells (Santiana et al., 2018; Altan-Bonnet et al., 2019). In fact, EVs derived from endothelial cells have been reported to transfer angiotensin-converting enzyme 2 (ACE-2) and contribute to the SARS-CoV-2 virus spreading (Wang et al., 2020). EVs can also modulate the inflammatory response triggered by virus infection. For example, rhinovirus infection of human bronchial epithelial cells results in the release of EVs containing tenascin-C, an immunomodulatory ECM protein which has the ability to induce inflammatory cytokine production (Mills et al., 2019).

Altogether, EVs seem to be a relevant mechanism in the pathogenesis of ARDS since they can activate inflammation, alter the alveolar capillary barrier and lead to alveolar edema in the lung (the summary of the main effects induced by EVs in ARDS is provided in Table 2). After lung injury, the release of lung-derived EVs into the systemic circulation also might contribute to damage in distal organs. Finally, we should consider that EVs derived from BALF, although less accessible than those EVs from serum/plasma, might reflect better the complete status of the lung microenvironment and be more useful to elucidate the pathophysiology of ARDS (Thompson et al., 2017).

**TABLE 2:** Contribution of EVs to the pathogenesis of ARDS.

*In vitro* models			

Source (Cell/Fluid/Tissue)	EV type	Effects	References
**Macrophages**	MVs and apoptotic bodies	Monocyte differentiation into macrophages (EV cargo: miR-223) and epithelial cell growth promotion (EV cargo: mir-221 and miR-222).	Ismail et al., 2013; Zhu et al., 2017
	EVs	Endothelial inflammation (via NFκB activation), endothelial barrier disruption (via VCAM-1, ICAM-1 and E-selectin upregulation), exacerbation of endothelial thrombogenicity (via TF EV-mediated transfer), and endothelial apoptosis (via p20 EV-mediated transfer).	Aharon et al., 2008; Wang et al., 2011; Mitra et al., 2015
**Endothelial cells**	EVs	Monocyte adhesion and recruitment via upregulating IL-6, IL-8, CXCL-1, MCP-1, CCL4, and CCL5 (EV cargo: CXCL-10 and CCL-5).	Hosseinkhani et al., 2018
	MVs	Upregulation of ICAM-1 expression mediated by activation of EGFR and PARP-1. Caveolae-dependent mechanism.	Andrews and Rizzo, 2016
**Neutrophils**	MVs	Antimicrobial effect (EV cargo: CR1, MPO and elastase).	Hess et al., 1999
	EVs	Anti-inflammatory effects (via decreasing IL-6, IL-8, IL-10, IL-1β, TNF-α, and CXCL-1, and TGF-β enhancement) and reduction of alveolar permeability (via PAR-1 inhibition) (EV cargo: miR-223, miR-126, miR-150, miR-451a).	Gasser and Schifferli, 2004; Eken et al., 2010, 2013; Neudecker et al., 2017; Youn et al., 2021
	EVs	Macrophage inflammation (EV cargo: miR-1260, miR-1285, miR-4454, miR-7975).	Youn et al., 2021
	EVs	Endothelial cell activation via increasing TF, ICAM-1, MCP-1, IL-6, and IL-8 production.	Mesri and Altieri, 1998, 1999
	EVs	Oxidative stress induction on endothelial cells (via MPO EV-mediated transfer) and platelets (via arachidonic acid EV-mediated transfer).	Pitanga et al., 2014
	EVs	Vascular permeability increase (EV cargo: cathepsin, S100A-8, S100A-9).	Dalli et al., 2013
	Exosomes	ECM degradation (via elastase EV-mediated transfer).	Mammoto et al., 2013; Genschmer et al., 2019
**Platelets**	EVs	Monocyte activation and recruitment (via increasing CD11b, LFA-1 and Mac-1), and oxidative stress induction on monocytes and endothelial cells (via arachidonic acid EV-mediated transfer).	Barry et al., 1997; Nomura et al., 2001
	EVs	Vascular permeability via inflammasome activation (EV cargo: IL-1β) and increased endothelial adhesiveness via ICAM-1 upregulation (EV cargo: miR-320b and CCL5).	Nomura et al., 2001; Mause et al., 2005; Gidlof et al., 2013; Hottz et al., 2013
	EVs	Endothelial cell apoptosis via repressing BCL2L1 and BCLAF1 genes (EV cargo: miR-142-3p).	Bao et al., 2017
***E. coli* induced-ALI in *ex vivo* human lungs**	Lung perfusate EVs	Pulmonary edema, impaired of fluid clearance, neutrophilic infiltration, and elevated concentrations of TNF-α in BALF.	Liu et al., 2019

***In vivo* models**			

**Model**	**EV type and source**	**Effects**	**References**

**LPS-induced ALI in mice**	BALF-derived	Inflammasome activation and induction of IL-1β production in macrophages (EV cargo: miR-466g, miR-466m-5p, miR-155, and miR-146a).	Shikano et al., 2019
		Inflammation and ECM degradation via induction of MMP1 and IL-6 production (EV cargo: CCN1).	Shi et al., 2018
		Macrophage derived-EVs, mainly produced by infectious stimuli, induced inflammation in macrophages via TLR6.	Lee et al., 2018b
	BALF-derived exosomes	Inflammation induction and alteration of TJs in alveolar epithelial cells (EV cargo: miR-155 and miR-146a).	Yuan et al., 2018
		ICAM-1, IL-8 and MCP1 upregulation in alveolar epithelial cells (EV cargo: TNF-α, IL-1β, and IL-6).	Soni et al., 2016; Zhang D. et al., 2019
	Endothelial cell-derived MVs	Neutrophil recruitment and increases in IL-1β and MPO in BALF.	Buesing et al., 2011; Li et al., 2015
		Impairment in vasodilatation via eNOS activation and reducing levels of NO.	Densmore et al., 2006
**ALI induced by sterile stimuli (oxidative stress, acid aspiration or mechanical ventilation) in rodents**	BALF-derived EVs	Epithelium derived-EVs, mainly produced by sterile stimuli, induced inflammation in macrophages via TLR2 activation.	Lee et al., 2018b
	BALF-derived MVs	Macrophage activation via MMP9, and TNF-α production and NFκB activation in macrophages (EV cargo: miR-320a, miR-22, miR-342), and macrophage migration through integrin β1 expression (EV cargo: miR-17 and mir-221).	Lee et al., 2017
	Epithelial cell-derived EVs Endothelial cell-derived MVs	Macrophage inflammation via induction of IL-6, TNF-α, and MIP-2 production (EV cargo: caspase-3).	Moon et al., 2015
		Increased levels associated with pulmonary edema, inflammatory infiltrates, deterioration of gas exchange following ventilator-induced lung injury.	Cabrera-Benitez et al., 2015; Pan et al., 2017
**Sepsis-induced ALI in mice**	Alveolar epithelial cell-derived exosomes	Macrophage activation via NFκB (EV cargo: miR-92a-3p).	Liu et al., 2021
	Endothelial cell-derived MVs	Endothelial permeability (via MLC and VE-cadherin phosphorylation) and neutrophil activation (via CD11b overexpression) and NETs formation (EV cargo: c-Src kinase).	Chatterjee et al., 2020a

**Clinical studies**			

**ARDS patients**	BALF-derived EVs	Inflammation and ECM degradation via induction of MMP1 and IL-6 production (EV cargo: CCN1).	Shi et al., 2018; Morrell et al., 2020
	BALF-derived exosomes	Hydrolysis of lung surfactant phospholipids and inflammation induction (EV cargo: sPLA2).	Papadopoulos et al., 2020
**Cystic fibrotic patients**	BALF-derived EVs	Neutrophil chemotaxis and recruitment into alveolar space (EV cargo: S100A).	Useckaite et al., 2020

*Summary of the effects induced by EVs in experimental models of ALI. The type of EV described in each study is specified in the second column and is included only for comparison purposes. Please, note that current methods are unable to separate these vesicles efficiently.*

*ALI, acute lung injury; Ang-1, angiopoetin-1; ARDS, acute respiratory distress syndrome; BALF, bronchoalveolar lavage fluid; BCL2L1, B-cell lymphoma 2-like protein 1; BCLAF1, B-cell lymphoma 2-associated transcription factor 1; CCL, chemokine (C-C motif) ligand; CCN1, cellular communication network factor 1; CD, cluster of differentiation; c-Src, cellular Src; CXCL, chemokine (C-X-C motif) ligand; ECM, extracellular matrix; eNOS, endothelial nitric oxide synthase; EV, extracellular vesicles; HGF, hepatocyte growth factor; ICAM-1, intercellular adhesion molecule 1; IL, interleukin; LFA-1, lymphocyte function-associated antigen 1; LPS, lipopolysaccharide; Mac-1, macrophage-1 antigen or macrophage integrin; MCP-1, monocyte chemotactic protein 1; MIP-2, macrophage inflammatory protein 2; MLC, myosin light chain; MMP, matrix metalloprotease; MPO, myeloperoxidase; NETs, neutrophil extracellular traps; NFκB, nuclear factor kappa B; NO, nitric oxide; MCP-1, monocyte chemotactic protein 1; PAR-1, protease-activated receptor 1; sPLA2, secretory phospholipase A2; TGF-β, transforming growth factor β; TF, tissue factor; TJs, tight junctions; TLR, toll-like receptor; TNF-α, tumor necrosis factor α; VCAM-1, vascular cell adhesion molecule 1; VE-cadherin, vascular endothelial-cadherin.*

### Effects of Epithelial Cell-Derived Extracellular Vesicles

Lung epithelial cells are the primary source of pulmonary EVs in BALF (Kesimer and Gupta, 2015; Lee et al., 2017). In physiological conditions, epithelial EVs carry mucins and glycoproteins on their surface, suggesting their protective role in the innate mucosal defense of airways and mucus barrier maintenance (Kesimer et al., 2009). In lung diseases, epithelial EVs regulate pulmonary inflammation, including activation of immune cells (Whitsett and Alenghat, 2015). In mice with LPS-induced ALI and in ventilated ARDS patients, increased levels of cellular communication network factor 1 (CCN1) were found in BALF (Shi et al., 2018; Morrell et al., 2020). Secreted CCN1 during ALI can interact with integrins and WNT receptor of nearby epithelial cells and induce the secretion of IL-6 and MMP-1 through PI3K/Akt signaling (Shi et al., 2018), contributing to pulmonary inflammation and degradation of ECM (Lu et al., 2011). In agreement with evidence mentioned above, CCN1 was also shuttled in epithelial cell-derived EVs following cigarette smoke extract exposure and promoted the secretion of IL-6 and MMP-1 by nearby epithelial cells (Moon et al., 2014). These findings suggest the inherent effect of epithelial cell derived-EVs on epithelial barrier disruption during lung disease.

Epithelial cell-derived EVs also interact with immune cells, especially macrophages. Epithelial-derived EVs transfer their proinflammatory cargo, mainly to AM, resulting in macrophage and neutrophil activation and migration into the lung. In the hyperoxia-induced ALI animal model, epithelial cells release EVs enriched in caspase-3, which are internalized by macrophages, triggering the secretion of pro-inflammatory molecules such as TNF-α, IL-6, and macrophage inflammatory protein 2 (MIP-2) through activation of Rho-associated protein kinase 1 (ROCK1) (Moon et al., 2015). In this regard, ROCK signaling has been demonstrated to increase alveolar-capillary barrier permeability during ARDS through the modulation of cell adhesion molecules such as ZO-1, filamentous actin (F-actin), or ICAM-1 (Abedi et al., 2020). Furthermore, epithelial-derived EVs from hyperoxia-induced ALI upregulate TLR2, Myd88, TNF-α, and IL-6 in AM, all of them activators of NFκB signaling (Lee et al., 2018b). On the other hand, EVs derived from AECs from both cystic fibrosis patients and *in vitro* assays contain S100A protein, which promotes neutrophil chemotaxis and recruitment to alveolar space (Useckaite et al., 2020).

Regarding miRNAs, several studies show the delivery of EV-miRNAs from epithelial cells to macrophages during ARDS/ALI. In acid-induced ALI conducted in animals, the elevated levels of miR-17 and mir-221 in epithelial cell derived-EVs cause macrophage migration upon expression of integrin β1 onto the macrophage surface (Lee et al., 2017). Levels of miR-320a, miR-22, and miR-342 are also increased in epithelial cell-derived EVs in hyperoxia-induced ALI. These EVs induce macrophage migration through the upregulation of MMP9, increased macrophage secretion of TNF-α, and NFκB activation, exacerbating the inflammatory response (Lee et al., 2017). Moreover, in a sepsis-induced ALI in mice, EVs derived from epithelial cells contained increased levels of miR-92a-3p that is transferred to AM and triggered NFκB activation (Liu et al., 2021). In the lung, the activation of NFκB by TNF-α has been described to downregulate tight junction proteins, such as claudin (CLDN)-2, CLDN4, CLDN5, and ZO-1 in the alveolar epithelium, resulting in increased alveolar permeability (Wittekindt, 2017).

Extracellular vesicles derived from AECs can also promote coagulation, and thus contribute to the formation of microthrombi in the lung and even the development of DIC. In this line, it is known that AECs release EVs containing tissue factor (TF) following a pro-inflammatory stimulus in ARDS patients (Kulshreshtha et al., 2013). TF is considered an initiator of the coagulation cascade, whose levels are increased in the lung of ARDS patients (Levi et al., 2003) and may result in excessive thrombin formation and the subsequent fibrin generation and deposition into the airspace in the lung (Bastarache et al., 2009). Thrombin has been reported to alter the permeability of alveolar epithelial and endothelial barriers by actin cytoskeleton remodeling through activating myosin light chain (MLC) kinase and Rho kinase signaling pathways, inducing a contractile tension that impaired alveolar-capillary barrier integrity in the injured lung (Trepat et al., 2005; Gavara et al., 2006; Hayashi et al., 2006). Although thrombin alters the endothelial barrier function, its effect on epithelial barrier is not completely understood. In this regard, Kawkitinarong et al. (2004) showed that the exposure of epithelial cell culture (A549) to thrombin enhance the epithelial barrier integrity via actin remodeling, elongating ZO-1 aggregates and accumulating both ZO-1 and occludin on the epithelial cell membrane. This effect was proposed as a compensatory mechanism of barrier restoration against lung injury (Kawkitinarong et al., 2004).

Taken together, the studies indicate that the epithelial-derived EVs contribute to ALI via activation of inflammatory responses and modulation of the alveolar-capillary permeability in the lung.

### Effects of Macrophage-Derived Extracellular Vesicles

The crosstalk between innate immune and epithelial cells is essential to maintain lung homeostasis. In healthy conditions, AM constitutively secrete EVs with suppressors of cytokine signaling proteins, such as SOCS, that are internalized by AECs and inhibit the inflammatory STAT pathway (Bourdonnay et al., 2015). In pathological conditions, however, macrophage-derived EVs significantly contribute to lung injury, mainly through activation of inflammation. Experimental models of LPS-induced ARDS show that AM-derived EVs are the dominant population in BALF (Soni et al., 2016; Zhang D. et al., 2019). Macrophage-derived EVs are enriched in TNF-α, IL-1β and IL-6, and upregulate ICAM-1, IL-8, and monocyte chemotactic protein (MCP)-1/CCL2 in AECs (Neri et al., 2011; Soni et al., 2016). Also, activated macrophages release EVs with miRNAs that differentiate naive monocytes into macrophages (miR-223; Ismail et al., 2013), or promotes epithelial cell growth (miR-221 and miR-222; Zhu et al., 2017) in cultured cells.

The EV-mediated communication between macrophages and endothelial cells has also been reported. Macrophages activated by *Mycobacterium tuberculosis* infection release EVs that activate NFκB and the Type 1 interferon pathways in endothelial cells (Li et al., 2018). The activation of NFκB in endothelial cells has been found to upregulate leukocyte adhesion molecules and increase endothelial barrier permeability (Sawa et al., 2008). Accordingly, the incubation of endothelial cells with EVs from a human monocyte cell line (THP-1) pre-incubated with LPS results in the activation of ERK1/2 and NFκB signaling pathways, and the expression of the endothelial-leukocyte adhesion proteins VCAM-1, ICAM-1, and *E*-selectin. These proteins mediate the adhesion of leukocytes to the endothelium, increase the endothelial barrier permeability and trigger endothelial apoptosis (Aharon et al., 2008; Wang et al., 2011). These EVs from human monocytes (THP-1) challenged with LPS also contain increased levels of active caspase-1 (p20) capable of inducing apoptosis in human pulmonary microvascular endothelial cell (HPMEC) *in vitro* (Mitra et al., 2015).

Monocyte-derived EVs have been reported to play a role in activating coagulation during sepsis. Monocyte-derived EVs express on their surface pro-coagulant molecules, such as PS or TF, which activate both extrinsic and intrinsic coagulation pathways (Iba and Ogura, 2018). In this line, the presence of TF on monocyte-EVs has been associated with the development of DIC (Hellum et al., 2014; Delabranche et al., 2016). The co-incubation of monocyte-derived EVs with endothelial cells increases TF and decreases the anticoagulant tissue factor pathway inhibitor (TFPI) and thrombomodulin levels in endothelial cells (Aharon et al., 2008). Therefore, monocytes not only activate coagulation but also they are capable of diminishing the anticoagulant properties of the vascular luminal surface of endothelial cells, thus creating a pro-thrombotic intravascular environment.

### Effects of Endothelial Cell-Derived Extracellular Vesicles

Endothelial cells are the main regulator of vascular homeostasis, modulating vascular tone, inflammation, coagulation, and angiogenesis (Chatterjee et al., 2020a). One of the characteristic features of ARDS is the presence of endothelial disruption, which can be caused by a variety of stimuli, such as mechanical stretch, cytokines, thrombin, or infection (Thompson et al., 2017). The activation of endothelial cells by some of the stimuli mentioned above results in endothelial cytoskeleton rearrangement, secretion of pro-inflammatory molecules, upregulation of endothelial adhesion molecules, and/or apoptosis. The activation of endothelial cells by some of the stimuli mentioned above results in endothelial cytoskeleton rearrangement, secretion of pro-inflammatory molecules rearrangement, secretion of pro-inflammatory molecules and/or upregulation of adhesion molecules, such as *E*-selectin, *P*-selectin, ICAM-1, VCAM-1, or PECAM-1 which facilitate the adhesion and transendothelial migration of leukocytes to sites of injury (Vassiliou et al., 2020).

All these events contribute to lung injury and facilitate the infiltration of immune cells into the alveolar space, exacerbating the lung damage (Millar et al., 2016; Vassiliou et al., 2020). EVs released from activated endothelial cells have been reported to promote the dysfunction of the alveolar-capillary barrier in ARDS. In particular, the administration of endothelial cell derived-EVs to mice increased levels of IL-1β, myeloperoxidase (MPO), and neutrophil recruitment in BALF. Interestingly, these effects are similar to those observed in LPS-mediated lung injury models (Buesing et al., 2011; Li et al., 2015a). In injured lungs, IL-1β increases the alveolar epithelial and endothelial permeability via Rho-activation. Specifically, the activation of Rho phosphorylates β-catenin and induces the stress actin fiber formation, increasing intercellular gaps (Ganter et al., 2008). Accordingly, in experimental models of VILI, elevated levels of endothelial cell derived-EVs in plasma were associated with higher lung edema score and worse gas-exchange (Cabrera-Benitez et al., 2015; Pan et al., 2017). Moreover, nitrated sphingosine-1-phosphate receptor 3 (S1PR3), a critical molecule mediating vascular permeability via Rho activation, was found within EVs released by lung endothelial cells exposed to barrier disruptive agents (such as LPS or mechanical stress) and association between increased S1PR3 plasma concentration and mortality was further validated in ARDS patients (Sun et al., 2012).

Endothelial cell-derived EVs can affect the endothelial barrier function by directly targeting the vascular endothelium. Increased levels of c-Src kinase in endothelial cell derived-EVs impair adherens junction integrity and cytoskeleton homeostasis of targeted endothelial cells through the phosphorylation of MLC and vascular endothelial-cadherin (VE-cadherin) (Chatterjee et al., 2020a, b). In experimental models of ALI, the administration of endothelial cell-derived EVs attenuates the activation of endothelial nitric oxide synthase (eNOS), reducing the levels of NO and impairing vasodilation (Densmore et al., 2006). Mouse lung endothelial cells challenged with TNF-α release EVs enriched in caveolin-1, which upregulates ICAM-1 expression and activates protease-activated receptor-1 (PAR-1) in an EGFR/NFκB-dependent manner (Andrews and Rizzo, 2016). The activation of PAR-1 has been reported to phosphorylate MLC and consequently generate F-actin fibers, resulting in endothelial cell contraction and barrier permeability (Grimsey and Trejo, 2016).

Endothelial cell-derived EVs also target and activate macrophages. TNFα-treated endothelial cells (HUVEC) release EVs enriched in several pro-inflammatory mediators such as chemokine (C-X-C motif) ligand 10 (CXCL-10), and chemokine (C-C motif) ligand 5 (CCL-5). The incubation of these EVs in a monocyte cell culture (THP-1) elevate the expression of key chemotactic mediators, such as IL-6, IL-8, CXCL-10, MCP-1/CCl2, and the macrophage inflammatory proteins CCL-4 and CCL-5, increasing monocyte adhesion and mobilization to the endothelium (Hosseinkhani et al., 2018).

Neutrophil activation mediated by endothelial cell-derived EVs has also been reported. In mice with abdominal sepsis induced by cecal ligation and puncture, the activated endothelial cells release EVs that upregulate the expression of CD11b on neutrophils and trigger the formation of neutrophil extracellular traps (NETs) (Chatterjee et al., 2020a, b). NETs are structures with antimicrobial effects composed of DNA complex with MPO or citrullinated histones which are developed in response to infectious stimuli (Rada, 2019). However, the excessive production of NETs causes epithelial and endothelial damage by inducing pro-inflammatory responses (Sabbione et al., 2017), cell death (Saffarzadeh et al., 2012), and direct alterations on endothelial and epithelial barrier mainly by decreasing the levels of ZO-1, VE-cadherin, and β-catenin (Meegan et al., 2017; Ma et al., 2019; Surolia et al., 2021). In addition, increased plasma levels of NETs in humans have been associated with ARDS severity and mortality (Lefrançais et al., 2018).

Endothelial cell-derived EVs also participate in coagulation. In cultured endothelial cells, TNF-α activates coagulation via TF/factor VII pathway and enhances the expression of ICAM-1, *E*-selectin, and PECAM-1 (Combes et al., 1999). This activation of coagulation in recipient endothelial cells initiates the assembly of clotting factors and leads to thrombin generation (Combes et al., 1999; Suades et al., 2012). As mentioned above, thrombin also induces endothelial cell contraction, favors the formation of intercellular gaps, and enhances endothelial barrier permeability (Kawkitinarong et al., 2004). Several studies also reported the association between endothelial cell-derived EVs and DIC (Delabranche et al., 2013; Alhamdi and Toh, 2016), supporting the notion that endothelial cell-derived EVs play a major role in coagulopathies. In fact, elevated levels of circulating TF in endothelial cell-derived EVs correlate with DIC score in patients with sepsis (Matsumoto et al., 2015) and have been associated with severity and mortality in patients with influenza A infection (Rondina et al., 2016) and COVID-19 (Rosell et al., 2021). Accordingly, increased levels of TF-EVs have been previously associated with mortality in severe influenza A infection (Rondina et al., 2016). However, the levels of EVs with surface anticoagulant antigens, such as thrombomodulin and endothelial protein C receptor, are also elevated in septic patients with DIC (Matsumoto et al., 2015). This suggests that the global effect of endothelial cell derived-EVs on coagulation may vary with the progression of the disease (Iba and Ogura, 2018).

### Effects of Neutrophil-Derived Extracellular Vesicles

Neutrophils, along with macrophages, are key mediators in inflammatory responses (Hong, 2018). Resting and activated neutrophils have been shown to release EVs with different functional properties, such as anti-inflammatory, pro-inflammatory, antibacterial or procoagulant effects (Hong, 2018; Kolonics et al., 2020). These contradictory effects seem to be influenced by the stimulus used for neutrophil activation, the EV isolation procedures or the environment of the target cell, among others (Hong, 2018; Kolonics et al., 2020).

The pro-inflammatory effects of neutrophils infiltrated into alveolar space during ARDS have been widely described (Thompson et al., 2017; Mikacenic et al., 2018). However, neutrophils activated with the bacterial peptide formyl-methionine-leucine-phenylalanine (fMLP) release EVs with antimicrobial effect expressing the opsonin complement receptor 1 (CR1) and other antimicrobial proteins, such as MPO and elastase (Hess et al., 1999). EVs derived from fMLP-activated neutrophils also exert protective effects in a VILI mouse model by direct transferring miR-223 to AECs, reducing alveolar permeability and the release of inflammatory cytokines (IL-6, IL-1β, CXCL1) via PAR-1 inhibition (Neudecker et al., 2017). The integrin CD11b, harbored on the surface of neutrophil-derived EVs, has been proposed as responsible for the binding of these EVs to epithelial cells (Slater et al., 2017).

The surface of neutrophil-derived EVs can contain phosphatidylethanolamine and PS that interact with Mer tyrosine kinase receptor (MerTKR) of macrophage surface (Eken et al., 2010, 2013). The incubation of activated macrophages (M1-like phenotype) with neutrophil-derived EVs reduces the production of pro-inflammatory molecules (IL-6, IL-8, IL-10, and TNF-α) and enhances the release of the anti-inflammatory transforming growth factor β (TGF-β) from activated macrophages (Gasser and Schifferli, 2004; Eken et al., 2010, 2013). Youn et al. (2021) described two different subpopulations of neutrophil-derived EVs with opposite effects on macrophage polarization. During migration to inflamed tissue, neutrophils attach to endothelium and develop elongated uropods. In these conditions, neutrophils release EVs containing pro-inflammatory miRNAs such as miR-1260, miR-1285, miR-4454, and miR-7975. These miRNAs exert a proinflammatory effect on macrophages and induce macrophage polarization from M0 to M1-proinflammatory phenotype. However, non-migrating neutrophils release EVs enriched in anti-inflammatory miRNAs, such as miR-126, miR-150, and miR-451a, which promote the macrophage polarization to M2-anti-inflammatory phenotype (Youn et al., 2021).

Neutrophil-derived EVs are also capable of targeting endothelial cells through the integrins CD66b, LFA-1 (CD11a/CD18) and Mac-1 (CD11b/CD18) harbored on their EV surface (Gasser and Schifferli, 2004). Like macrophage-derived EVs, neutrophil-derived EVs exert pro-inflammatory effects on endothelial cells. Specifically, fMLP-stimulated neutrophils produce EVs that activate the c-Jun N-terminal kinase (JNK) pathway in endothelial cells and increase the production of TF, the adhesion molecule ICAM-1, the chemokine MCP-1/CCl2, and the cytokines IL-6, IL-8 (Mesri and Altieri, 1998, 1999), revealing the direct effect of neutrophil derived-EVs on leukocyte adhesion and coagulation in endothelial cells. Moreover, EVs released by neutrophils activated by calcium ionophore (A23187) induce oxidative stress on endothelial cells by transferring MPO (Pitanga et al., 2014), an enzyme that produces the oxidant hypochlorous acid (HOCl), which has an important role in endothelial cell death and thrombogenicity (Sugiyama et al., 2004). Neutrophil-derived EVs also contribute to the endothelial barrier disruption by degrading the ECM. During degranulation, neutrophils release EVs enriched in neutrophil elastase on their surface, which contributes to the degradation of multiple ECM components (Mammoto et al., 2013; Genschmer et al., 2019).

Other pro-inflammatory molecules involved in vascular permeability, such as cathepsin G, S100A-8, and S100A-9 have also been found in EVs from fMLP-stimulated neutrophils (Dalli et al., 2013). Cathepsin G also induces endothelial permeability since it causes F-actin rearrangement and detachment of the plasminogen activator inhibitor-1 from the subendothelial matrix (Iacoviello et al., 1995). In addition, cathepsin G can degrade VE-cadherin and impair junction integrity (Cohen-Mazor et al., 2014). S100A8 and S100A9 increase endothelial permeability via binding to receptors TLR4 and RAGE and then activating p38 and ERK1/2 pathways, resulting in ZO-1 and F-actin disassembly (Wang et al., 2014). In this regard, S100A8 was upregulated in the blood and lung of SARS-CoV-2-infected animals and patients, and it was associated with the increase of recruited neutrophils in the lung (Guo et al., 2021). On the contrary, beneficial effects of neutrophil-derived EVs on endothelial cells have also been reported. In fact, fMLP-stimulated neutrophils produce anti-inflammatory EVs containing annexin 1, which reduces the neutrophil-endothelial adhesion *in vitro* and neutrophil infiltration *in vivo* (Dalli et al., 2008).

Neutrophil-derived EVs also activate platelets. For instance, EVs from fMLF-stimulated neutrophils transfer arachidonic acid to platelets via Mac-1 and clathrin, increase the synthesis of thromboxane by platelets, and induce oxidative stress *in vivo* (Rossaint et al., 2016).

### Effects of Platelet-Derived Extracellular Vesicles

Experimental models of ALI have evidenced the role of platelets in the pathogenesis of ARDS, mainly through their interaction with endothelial and immune cells. Specifically, platelets are involved in the dysregulation of coagulation, and they also contribute to the excessive inflammation and alveolar-capillary disruption occurring in ARDS (Yadav and Kor, 2015; Chatterjee et al., 2020b).

The presence of *P*-selectin and CD40 on the surface of platelet-derived EVs is responsible for their binding to immune and endothelial cells (Heijnen et al., 1999; Forlow et al., 2000; Kuravi et al., 2019). Platelet-derived EVs activate monocytes (THP-1 culture) and endothelial cells (HUVEC) and modify their adhesiveness (Nomura et al., 2001). Platelet-derived EVs increase the expression of CD11b, LFA-1, and Mac-1 on monocytes and their production of cytokines (IL-8, IL-1β) (Barry et al., 1997; Nomura et al., 2001). In experimental models of ALI, the platelet-neutrophil interaction, crucial for the alveolar neutrophil recruitment, seems to be partially mediated by platelet secretion of CCL5, CXCL4, and *P*-selectin (Grommes et al., 2012). In addition, platelet-derived EVs also transfer arachidonic acid to both endothelial and monocyte cells, enhancing the cyclooxygenase 2 (COX-2) activity and therefore exacerbating the oxidative stress (Barry et al., 1997).

Platelet-derived EVs also enhance the cytokine production of endothelial cells, specifically IL-6, IL-8, and IL-1β (Nomura et al., 2001). In addition, platelet-derived EVs are enriched in IL-1β, whose transport to endothelial cells increases vascular permeability via activation of the NLRP3-inflammasome pathway (Hottz et al., 2013). Furthermore, EVs from platelets upregulate ICAM-1 on endothelial cells via miR-320b (Gidlof et al., 2013), resulting in increased adhesion between endothelial and immune cells (Nomura et al., 2001; Gidlof et al., 2013). EVs derived from activated platelets contain substantial amounts of CCL5 and facilitate the transfer of CCL5 to endothelial cells, triggering the recruitment of immune cells (Mause et al., 2005). Increased activation and adhesion of immune cells on endothelial cells may result in extravasation and additional activation of vessels, contributing to vascular inflammation and permeability. Moreover, platelet-derived EVs induce apoptosis of endothelial cells by the action of miR-142-3p and the subsequent repression of Bcl-2 like 1 (BCL2L1) and Bcl-2-associated transcription factor (BCLAF1) genes (Bao et al., 2017). Therefore, the increased levels of circulating platelet-derived EVs have been strongly proposed as indicators of endothelial dysfunction (Chiva-Blanch et al., 2019; Oggero et al., 2019; de Freitas et al., 2021).

To the best of our knowledge, direct interactions between platelet-derived EVs and alveolar epithelium have not been reported yet. Several studies have demonstrated the enrichment of P-selectin and CD40 on the surface of platelet-derived EVs (Heijnen et al., 1999; Forlow et al., 2000; Kuravi et al., 2019). Although *P*-selectin expression is usually associated with endothelial and immune cells (Celi et al., 1997; Forlow et al., 2000), the expression of P-selectin in AECs was observed in the autopsy of SARS-CoV-1-infected patients and, as well as, in AECs cultured upon exposure to the SARS-CoV-1 (Yen et al., 2006), suggesting a possible interaction between epithelial cells and platelet-derived EVs.

Platelet-derived EVs have been mainly defined as procoagulant agents in pathological conditions, thus contributing to the link between inflammation and thrombosis (Nieuwland et al., 2000; Tripisciano et al., 2017; Balbi et al., 2021; Puhm et al., 2021). Platelet-derived EVs contained high levels of TF and PS (Nieuwland et al., 2000; Tripisciano et al., 2017). PS expressed on the surface of the platelet-derived EVs is a catalytic site for the assembly of coagulation complexes, enhancing thrombin formation in a factor XII-dependent manner (Tripisciano et al., 2017). During SARS-CoV-2 infection, substantial amounts of procoagulant platelet-derived EVs containing TF were released to blood circulation, compared with non-infected patients (Balbi et al., 2021). These EVs have been proposed to act as clotting initiation agents, contributing to the severity of this disease. In fact, several studies consider the circulating platelet-derived EVs as a hallmark of SARS-CoV-2 infection (Balbi et al., 2021; Cappellano et al., 2021; Guervilly et al., 2021).

## Protective Role of Endogenous Extracellular Vesicles

As discussed in the previous section, there is growing evidence that EVs play an important role in the pathogenesis of ARDS. However, there is also some evidence suggesting that some endogenous EVs may also have a protective effect in ARDS patients (Mahida et al., 2020a). For example, some clinical studies have reported that total leukocyte-derived EVs are associated with a better prognosis in patients with sepsis (Lashin et al., 2018) or ARDS (Guervilly et al., 2011; Mahida et al., 2020a, b). Notably, circulating leukocyte-derived EVs expressing α2-macroglobulin (A2MG) isolated from septic patients were shown to reduce endothelial cell permeability and increased bacterial phagocytosis by neutrophils *in vitro* (Lashin et al., 2018). Similarly, some studies have reported anti-inflammatory effects by EVs derived from neutrophils on AM and AECs (Gasser and Schifferli, 2004; Eken et al., 2010; Mahida et al., 2020a, b). Indeed, transfer of miR-223 by neutrophil-derived EVs to airway epithelial cells has been shown to reduce protein permeability and inflammatory cytokine release *in vitro* and *in vivo* (Neudecker et al., 2017). Finally, several studies have demonstrated that EVs from endothelial progenitor cells (EPCs) also reduce inflammation and lung injury in several models of ALI (Wu et al., 2018; Zhou et al., 2018, 2019).

## Therapeutic Potential of Extracellular Vesicles Derived From Mesenchymal Stem Cells in Acute Respiratory Distress Syndrome

Mesenchymal stem cells (MSCs) are multipotent progenitor cells that can be isolated from multiple tissues including bone marrow, adipose tissue, and umbilical cord tissue, blood and perivascular tissue. The source may impact the immunomodulatory effects, proliferation properties and therapeutic benefits of MSCs. Bone marrow-derived MSCs were the first type of MSCs isolated and are the most widely used for lung injury (Monsel et al., 2016; Mohammadipoor et al., 2018). Administration of MSCs induces potent anti-inflammatory and immunomodulatory effects and has been proven to decrease lung injury and increase survival in several preclinical models of ALI (Pati et al., 2011; Curley et al., 2012; Jackson et al., 2016; Xiao et al., 2020). Thus, MSCs were able to maintain the integrity of the lung microvascular barrier (Li C. et al., 2019) and reduce the formation of pulmonary edema (Gupta et al., 2007) in several models of endotoxin-induced ALI due to a down-regulation of pro-inflammatory cytokines, such as TNF-α or MIP-2, and an increase in the production of the anti-inflammatory cytokine IL-10 (Gupta et al., 2007). Moreover, MSCs also attenuated neutrophil-predominant inflammation and lung injury in an *in vivo* rat model of VILI (Lai et al., 2015). In Influenza mice models, MSCs also limited alveolar inflammation (Li et al., 2016) and prevented the downregulation of sodium and chloride transported in infected cells, reducing the impairment of alveolar fluid clearance and attenuating lung injury (Chan et al., 2016).

Despite these long-lasting therapeutic effects in a wide variety of *in vivo* experimental models, the engraftment rate of MSC has been shown to be extremely low (Monsel et al., 2016; Mastrolia et al., 2019). This unexpected low engraftment rate represented a major challenge in explaining the beneficial effects of MSCs (since in most cases, the cells were only temporarily resident in the host). Indeed, administration of MSC-derived conditioned medium (MSC-CM) recapitulates the therapeutic effects of MSCs in ARDS and other inflammatory lung diseases through activation of anti-inflammatory, pro-survival and anti-apoptotic pathways (Monsel et al., 2016; Byrnes et al., 2021). MSC-CM can mitigate the inflammatory response of the injured endothelium by preserving the integrity of the vascular barrier, restoring the normal status of membrane adhesion molecules β-catenin and VE-cadherin, and preventing inflammatory cell binding to endothelial cells (Pati et al., 2011). Moreover, it has been demonstrated the capability of MSC-CM to reduce the secretion of TNF-α by macrophages mediated by IL-1RA (Ortiz et al., 2007). The administration of MSCs and MSC-CM improved lung endothelial barrier integrity and the rate of alveolar fluid clearance in an *ex vivo* model of perfused human lungs injured by endotoxin. This effect was, at least partially, mediated by the release of keratinocyte growth factor (KFG) which contributes to restore sodium dependent alveolar fluid transport (Lee et al., 2009). Administration of MSC-CM also exerted protective effects following VILI, decreasing lung inflammation (as evidenced by the reduction in alveolar inflammatory cell counts, the decrease in IL-6 and TNF-α production or the increase in IL-10), improving systemic oxygenation and enhancing alveolar fluid clearance through a mechanism which may also be mediated through KGF secretion (Curley et al., 2012). Intratracheal administration of MSCs or MSC-CM also reduced the total number of infiltrated neutrophils in BALF and attenuated the formation of lung edema in mice with endotoxin-induced ALI (Ionescu et al., 2012). In contrast, in a rat model of *E. coli*-induced pneumonia administration of MSCs reduced the infiltration of neutrophils and the total amount of protein in BALF, whereas MSC-CM was less effective, improving animal survival but without significant mitigation of the severity of lung injury or inflammation (Devaney et al., 2015).

Overall, we now have a large body of evidence suggesting that MSCs act through paracrine effects, rather than (trans)differentiating and incorporating into the host tissue. In this regard, the discovery that EVs released by MSCs act as carriers of bioactive molecules (Maas et al., 2017), opens the possibility to develop new therapies based on the use of stem cells but cell-free and therefore potentially safer and amenable to standardization (Mohammadipoor et al., 2018; Lanyu and Feilong, 2019). In line with this, administration of MSCs-derived EVs (MSC-EVs) are also showing promising results in experimental models of ALI from different etiologies (Lee et al., 2019; Table 3). Accordingly, administration of human MSC-EVs to mice model of ALI induced by IT administration of endotoxin attenuated the influx of inflammatory cells, decreased the total protein content in BALF and reduced the extravascular lung water content (Zhu et al., 2014). In this study, Zhu et al. (2014) demonstrated that IT administration of MSC-EVs was able to reduce inflammation and restore the barrier function in an endotoxin-induced ALI model through a mechanism which involved KGF. In a later study, these authors confirmed that IV administration of MSC-EVs also improved survival and reduced lung protein permeability in a model of *E. coli*-induced pneumonia through a mechanism also mediated by KGF (Monsel et al., 2015). In addition, MSC-EVs were able to restore protein permeability in primary cultures of human alveolar epithelial type II cells, induce anti-inflammatory M2 polarization in a macrophage cell line (Zhu et al., 2014) and enhance bacterial phagocytosis by human monocytes (Monsel et al., 2015).

**TABLE 3:** Therapeutic potential of EVs derived from mesenchymal stem cells in ARDS.

*In vitro* models			

Model	EVs source	Main effects	References
Human macrophages stimulated with LPS or BALF from ARDS patients	Human BM-MSC	Decrease in inflammatory cytokines secretion and increase in M2 macrophage markers, IL-10 secretion and phagocytic capacity.	Zhu et al., 2014; Monsel et al., 2015; Morrison et al., 2017; Tang et al., 2017
Human endothelial cells stimulated with LPS, cytokines or plasma from ARDS patients	Human/mice BM-MSC	Increase in proliferation and IL-10 levels. Reduction in pulmonary capillary permeability, apoptosis, mitochondrial dysfunction and secretion of inflammatory cytokines and Ang-1 (EV cargo: HGF and mitochondria).	Tang et al., 2017; Wang et al., 2017; Hu et al., 2018; Dutra Silva et al., 2021
Human alveolar epithelial type 2 cells stimulated with LPS, cytokines or plasma from ARDS patients	Human BM-MSC	Decrease in protein permeability, inflammatory cytokines and Ang-1 secretion and mitochondrial dysfunction (EV cargo: mitochondria).	Monsel et al., 2015; Morrison et al., 2017; Dutra Silva et al., 2021
Human alveolar epithelial type 2 cells stimulated with *Influenza virus*	Swine BM-MSC	Reduction in replication and apoptosis.	Khatri et al., 2018
*Ex vivo* perfused human lungs rejected for transplantation	Human BM-MSC	Increase in alveolar fluid clearance and airway and hemodynamic parameters. Decrease in lung weight gain.	Gennai et al., 2015
*Ex vivo* perfused human lungs injured with severe *E. coli* pneumonia	Human BM-MSC	Increase in alveolar fluid clearance. Decrease in bacterial count, absolute neutrophil count and protein permeability.	Park et al., 2019
*Ex vivo* cultured human precision cut lung slices	Human BM-MSC	Attenuation of mitochondrial dysfunction and downregulation of TNF-α, IL-8 and RAGE (EV cargo: mitochondria).	Dutra Silva et al., 2021

***In vivo* models**			

**Model**	**EVs source**	**Main effects**	**References**

Endotoxin-induced ALI in mice	Human BM-MSC	Improvement in lung mitochondrial bioenergetics and decrease in BALF total protein and cell count.	Dutra Silva et al., 2021
		Reduction in the extravascular lung water and total protein levels in BALF, demonstrating a reduction in pulmonary edema and lung protein permeability. MVs also reduced the influx of neutrophils and macrophage inflammatory protein-2 levels in the BAL fluid (EV cargo: KGF mRNA).	Zhu et al., 2014
		Reduction in the influx of inflammatory cells in the injured alveoli, MIP-2 and albumin levels in BALF, pulmonary capillary permeability and histological injury (EV cargo: Ang-1 mRNA).	Tang et al., 2017
		Decrease in alveolar leukocytosis and protein leak, mitochondrial dysfunction and mortality and increase in surfactant secretion (EV cargo: mitochondria).	Islam et al., 2012
		Improvement in survival and decrease in histological severity, influx of inflammatory cells, cytokines, protein and bacteria (EV cargo: KGF).	Monsel et al., 2015
Hyperoxia-induced ALI in mice	Human UCB-MSC	Attenuation of impaired alveolarization and angiogenesis, increased cell death. Diminishment of activated macrophages and inflammatory cytokines secretion (EV cargo: VEGF).	Ahn et al., 2018
Haemorrhagic shock-induced ALI in mice	Human BM-MSC	Significant decrease in lung vascular permeability (via decreased activation of the cytoskeletal GTPase RhoA).	Potter et al., 2018
Traumatic-induced (weight-drop method) ALI in rats	Rat BM-MSC	Increase in survival and IL-10 level and decrease in oxidative stress, cell count, inflammatory cytokines secretion and protein in BALF (EV cargo: mitochondria).	Li Q.C. et al., 2019
Influenza virus-induced ALI in pigs	Swine BM-MSC	Reduction in infiltration of inflammatory cells to the lungs, thickening of alveolar walls and number of collapsed alveoli.	Khatri et al., 2018

*ALI, acute lung injury; Ang-1, angiopoetin-1; ARDS, acute respiratory distress syndrome; BALF, bronchoalveolar lavage fluid; BM-MSC, bone marrow-derived mesenchymal stem cells; EV, extracellular vesicles; HGF, hepatocyte growth factor; IL, interleukin; KGF, keratinocyte growth factor; LPS, lipopolysaccharide; RAGE, receptor for advanced glyc end products; TNF-α, tumor necrosis factor α; VEGF, vascular endothelial growth factor.*

The direct effects of MSC-EVs on activated macrophages have also been confirmed in human monocyte–derived macrophages in the presence of BALF from ARDS patients and in murine AM (Morrison et al., 2017). In this study, the authors found that EVs derived from MSCs can modulate macrophage polarization through a mechanism involving the transfer of functional mitochondria and upregulation of oxidative phosphorylation. Furthermore, administration of AM treated with MSC-EVs, but not control macrophages, protected against endotoxin-induced lung injury, significantly reducing the protein content and the total number of neutrophils in BALF (Morrison et al., 2017). Phinney et al. (2015) also demonstrated that mitochondrial transfer by MSC is facilitated by the simultaneous release of EVs containing microRNA which suppressed TLR signaling and desensitized macrophages to the ingested mitochondria. Using miRNA microarray analysis, the authors explored the content of MSC-derived EVs obtained from five human donors, identifying 156 microRNAs (45 upregulated; 111 downregulated) that differed in abundance between EVs compared with their parent MSCs. The EVs were enriched in miR-451, miR-1202, miR-630, and miR-638, whereas miR-125b and miR-21 showed the largest reduction in MSC-EVs (Phinney et al., 2015).

MSC-EVs also have protective effects on pulmonary structural cells. In a murine model of haemorrhagic shock, both MSCs and their EVs were shown to attenuate vascular permeability in injured lungs through inhibition of RhoA GTPase activity in lungs, but through differential activation of proteins and pathways (Potter et al., 2018). In a swine model of influenza virus-induced ALI, MSC-EVs were able to inhibit the replication of influenza virus in epithelial cells and to significantly reduce the infiltration of inflammatory cells to the lungs, the thickening of alveolar walls and the number of collapsed alveoli in infected swine (Khatri et al., 2018). MSC-EVs were also effective on reducing the H_2_O_2_-induced epithelial cell death *in vitro* and hyperoxia-induced lung injuries *in vivo*, such as impaired alveolarization and angiogenesis, increased cell death and inflammatory responses. These protective effects against hyperoxic lung injury were apparently mediated by the transfer of vascular endothelial growth factor (VEGF) contained within these vesicles into pericytes, AM and ATII cells (Ahn et al., 2018). In endotoxin-induced lung injury, the protective effects induced by MSC-EVs on pulmonary edema were mediated partly by transfer of KGF mRNA to airway epithelial cells and angiopoietin-1 (Ang-1) mRNA to endothelial cells (Zhu et al., 2014; Tang et al., 2017; Mahida et al., 2020b). In endothelial cells stimulated with endotoxin *in vitro*, MSC-EVs augmented the expression of endothelial intercellular junction proteins, reduced endothelial cell apoptosis and inhibited the production of inflammatory cytokines through a mechanism involving the hepatocyte growth factor (HGF) gene carried inside the vesicles (Wang et al., 2017). Increasing evidence suggest that MSC-EVs improve alveolar-capillary barrier properties through restoration of mitochondrial functions via mitochondrial transfer. In a model of severe pneumonia induced by *E. coli*, MSC-EVs also augmented intracellular ATP levels in injured AECs which is critical to restore fluid clearance and surfactant production (Monsel et al., 2015). Although the mechanism was not elucidated in this study, the presence of mitochondria is critical for EV ability to reduce lung injury and restore mitochondrial respiration in the lung tissue (Dutra Silva et al., 2021). Indeed, mitochondrial transfer by MSC-EVs has been shown to play a key role in the amelioration of lung injury by modulating the phenotype of macrophages (Jackson et al., 2016) or restoring bioenergetic functions in AECs, ECs, and *ex vivo* cultured precision cut lung slices (Islam et al., 2012; McVey et al., 2019; Dutra Silva et al., 2021). Another explanation for the barrier-enhancing effect of MSC-EVs can be attributed to an increased VE-cadherin expression and enhanced VE-cadherin and β-catenin interaction, supporting barrier integrity (Potter et al., 2018; Simmons et al., 2019). MSC-EVs restored protein permeability across human lung microvascular endothelial cells (HLMVECs) exposed to an inflammatory insult (a mixture of IL-1β, TNF-α, and interferon γ [IFN-γ]) in part by maintaining inter-cellular junctions and preventing actin stress fiber formation. Incorporation of MSC EVs into HLMVECs through the surface receptor CD44 was required for restoration of protein permeability. The therapeutic effect of MSC EVs was associated with the transfer of Ang-1 from the EVs to the injured HLMVECs with subsequent secretion of the anti-permeability factor (Hu et al., 2018).

MSC-EVs have been shown to restore alveolar fluid clearance and reduce edema in *ex vivo* perfused human lungs rejected for transplantation and those injured by bacterial pneumonia (Gennai et al., 2015; Park et al., 2019). Similarly, *E. coli*-induced pneumonia in mice was ameliorated by MSC EVs via the well-documented barrier-stabilizing and anti-inflammatory effects of KGF (Monsel et al.,. 2015; McVey et al., 2019). Prophylactic treatment with MSC-EVs increased survival in rats undergoing traumatic lung injury; inflammatory cytokines, infiltrating leukocytes and the degree of pulmonary edema were all reduced (Li Q.C. et al., 2019).

## Strategies for Improving the Therapeutic Efficacy of Mesenchymal Stem Cells

Although MSCs have an innate potential to induce and/or contribute to regeneration, this potential is now known to be greatly influenced by diverse extrinsic factors such as the tissue source of the MSCs, the health status and age of the MSCs donor, the batch/lot of serum used for the *in vitro* culture of the MSCs, passage number, oxygen concentration, and the presence/absence of a pro-inflammatory environment when the MSCs are infused. Indeed, after transplantation, MSCs must confront a harsh environment which limit their survival and compromise their ability to migrate toward damaged tissues leading to unsatisfactory therapeutic results. In the pursuit of strategies to enhance the therapeutic potential of MSCs, preconditioning strategies are gathering increasing attention. In this context, one of the major challenges in MSC-based therapies is to develop methods that mimic the injury environment, without compromising cell quality and function. Thus, currently explored *in vitro* preconditioning strategies include environmental stimuli (such as exposure to hypoxia), treatment with cytokines or pharmacological agents, physical factor preconditioning or genetic engineering (summarized in Table 4; Ferreira et al., 2018; Han et al., 2019; Gorgun et al., 2021; Rolandsson Enes et al., 2021).

**TABLE 4:** Strategies for improving the therapeutic efficacy of MSCs in ARDS/ALI preclinical models.

Preconditioning strategy		ARDS/ALI preclinical model	Effects	References
**Hypoxia**		**Endotoxin**	↓ Neutrophil influx ↓ TNF-α ↑ IL-10 level	Li et al., 2015
		**Bleomycin**	↑ Anti-apoptotic factors (HGF and Bcl-2) ↑ Antioxidative factors (catalase and HO-1) ↑ Proangiogenic factors (VEGF)	Lan et al., 2015
**Cytokines**	**IFN-γ**	***E. coli***	↑ Macrophages phagocytosis ↑ Capillary endothelial barrier function	Varkouhi et al., 2019
**TLR ligands**	**TLR4**	***E. coli***	↑ Survival ↓ Lung damage ↑ Bacterial clearance ↓ Influx of inflammatory cells in BALF ↓ MIP-2 in BALF ↓ Total level of protein in BALF	Gupta et al., 2018
	**TLR3**	***E. coli***	↑ Bacterial clearance ↑ Phagocytic activity	Monsel et al., 2015
		***E. coli***	↓ Lung protein permeability	Park et al., 2019
**Genetic engineering**	**CXCR4**	**Endotoxin**	↑ MSC homing to injured lung tissue ↓ Lung protein permeability ↓ TNF-α levels ↑ IL-10 levels ↓ Lung pathology score ↓ Wet/dry ratio ↓ Total protein content in BALF	Yang et al., 2015
	**IL-10**	***E. coli***	↓ Infiltrated neutrophils ↑ Phagocytic capacity ↓ Markers of structural lung injury	Jerkic et al., 2019
		**HCl**	↓ TGF-β1, FN and fibrinogen in BALF ↓ Inflammation scores and Ashcroft scores	Islam et al., 2019
	**sST2**	**Endotoxin**	↓ Lung airspace inflammation and vascular leakage ↑ Alveolar architecture	Martinez-Gonzalez et al., 2013
	**HGF**	**I/R**	↑ Oxygen saturation ↓ Lung edema	Chen et al., 2017
		**HCl**	↓ TGF-β1, FN and fibrinogen in BALF ↓ Inflammation scores and Ashcroft scores	Islam et al., 2019
		**Radiation**	↓ TNF-α, IFN-γ, IL-6 and intercellular adhesion molecule-1 level ↑ IL-10 level ↓ Fibrosis progress	Wang et al., 2013
	**Ang-1**	**Endotoxin**	↑ Anti-inflammatory effects ↓ Capillary endothelial barrier function	Mei et al., 2007; Xu et al., 2008
	**PGE receptor 2**	**Endotoxin**	↑ MSC homing to injured lung tissue ↓ Alveolar-capillary barrier permeability ↓ TNF-α and IL-1β level	Han et al., 2016
	**HO-1**	**Endotoxin**	↑ Survival ↓ Alveolar-capillary barrier permeability ↓ Inflammatory markers ↑ HGF, KGF and IL-10 levels in serum and lungs	Chen et al., 2019
	**P130/E2F4**	**Endotoxin**	↑ MSC homing to injured lung tissue ↑ Differentiation into AECs II ↓ Alveolar-capillary barrier permeability	Zhang X. et al., 2019
	**microRNA-30b-3p**	**Endotoxin**	↓ Histopathology ↓ Alveolar-capillary barrier permeability ↓ Neutrophil infiltration ↓ MPO activity ↓ Alveolar inflammation	Yi et al., 2019
	**miR-22-3p**	**Endotoxin**	↓ Lung inflammation and oxidative stress ↓ Epithelial and endothelial apoptosis	Zheng et al., 2021

*AEC, alveolar epithelial cell; ALI, acute lung injury; Ang-1, angiopoetin-1; ARDS, acute respiratory distress syndrome; BALF, bronchoalveolar lavage fluid; Bcl-2, B-cell lymphoma 2; CXCR, CXC chemokine receptor; FN, fibronectin; HGF, hepatocyte growth factor; HO-1, heme oxygenase-1; IFN-γ, interferon gamma; IL, interleukin; KGF, keratinocyte growth factor; MIP-2, macrophage inflammatory protein 2; MPO, myeloperoxidase; MSC, mesenchymal stem cell; PGE, prostaglandin E; sST2, soluble IL-1 receptor-like-1; TGF-β, transforming growth factor β; TLR, toll-like receptor; TNF-α, tumor necrosis factor α; VEGF, vascular endothelial growth factor.*

### Preconditioning of Mesenchymal Stem Cells

#### Hypoxia-Treated Mesenchymal Stem Cells

Mesenchymal stem cells are routinely cultured under ambient oxygen conditions (21% O_2_) despite the physiological environment in the bone marrow and other tissues is hypoxic, with oxygen tension ranging from 2 to 12% O_2_. Furthermore, following transplantation, MSC can confront severe hypoxic conditions in the ischemic tissues, even below 1% O_2_, which often results in apoptosis (Rosová et al., 2008). Accordingly, it is well documented that culture under hypoxic conditions can promote the proliferation of MSCs, inhibit apoptosis, and facilitate migration and chemotaxis thereby improving the effectiveness of MSCs in treating ARDS/ALI (Bader et al., 2015; Beegle et al., 2015; Wang et al., 2021).

Hypoxic preconditioning modulates the secretion of cytokines and other soluble factors enhancing the immunomodulatory properties of MSCs (Frazier et al., 2013; Roemeling-van Rhijn et al., 2013). For example, hypoxia markedly upregulated the expression of tryptophan-catabolizing enzyme indolamine 2,3 deoxygenase (IDO) or prostaglandin E (PGE) synthetase, two well-known activators of regulatory T cells (Tregs), in human MSCs (Nemeth et al., 2009; Engela et al., 2013; English et al., 2013; Roemeling-van Rhijn et al., 2013). Moreover, it has been reported that hypoxia stimulates the production of anti-inflammatory cytokines (Jiang et al., 2015) and induces a metabolic switch from oxidative phosphorylation to glycolysis (Contreras-Lopez et al., 2020). Hypoxic preconditioning also increases the angiogenic potential of MSCs (Leroux et al., 2010; Liu et al., 2015; Ferreira et al., 2018; Han et al., 2018). Thus, hypoxia has been shown to increase the release of several factors involved in the blood vessel formation such as VEGF, HGF, fibroblast growth factor 2 (FGF2), insulin-like growth factor 1 (IGF-1), Ang-1 or erythropoietin (Crisostomo et al., 2008; Kim et al., 2015). Notably, administration of EVs from MSCs cultured under hypoxic conditions has been shown to promote angiogenesis following acute myocardial infarction. Thus, injection of these EVs (80 μg of protein) thirty minutes after ligation of the coronary artery significantly reduced the infarct size and improved cardiac performance (Bian et al., 2014). Similarly, intravenous administration of hypoxia – preconditioned EVs (50 μg of protein) immediately after the initiation of reperfusion also demonstrated cardioprotective effects in ischemia/reperfusion (I/R) injured hearts (Park et al., 2018). Proteomic analysis of these EVs has identified the presence of Ang-1, VEGF, IGF, MCP-1, Tie-2/TEK, and IL-6 as potential mediators for these hypoxia-augmented proangiogenic effects (Chen et al., 2014). In line with these evidences, hypoxic preconditioning has been shown to increase the protective effects of MSC-EVs in experimental models of lung injury (Table 4). Thus, hypoxic preconditioning potentiated the therapeutic effects of MSC-EVs in a model of endotoxin-induced ALI, facilitating the reduction of neutrophil influx, the decrease of TNFα and the upregulation of IL-10 (Li et al., 2015). In line with this, acute lung transplantation of hypoxia-preconditioned MSCs also exerted better therapeutic effects in bleomycin-induced pulmonary fibrotic mice and enhanced the survival rate of engrafted MSCs, due to the increased expression of anti-apoptotic (HGF, Bcl-2), anti-oxidative (catalase, heme oxygenase 1), and proangiogenic (VEGF) factors (Lan et al., 2015).

#### Treatment of Mesenchymal Stem Cells With Cytokines

Another strategy proposed to improve the therapeutic potential of MSCs is preconditioning with inflammatory cytokines such as IFN-γ, TNF-α, or IL-1β. One of the most studied strategies is pre-treatment with IFN-γ, which plays an important role in the regulation of immunomodulatory functions of MSCs (Krampera et al., 2006; Polchert et al., 2008; Croitoru-Lamoury et al., 2011). Following preconditioning with IFN-γ, MSC show upregulation of IDO and PGE synthetase which results in increased suppression of natural killer cells and Th1 activation (Tipnis et al., 2010; Noone et al., 2013). Priming with IFN-γ also enhanced the ability of MSCs to induce a greater number of Tregs in comparison to control MSCs but by an IDO-independent pathway (Chinnadurai et al., 2014). IFN-γ, along with TNF-α, IL-1α, and IL-1β induces Gal-9 in MSC which also contribute to suppressing T-cell proliferation (Gieseke et al., 2013). Preconditioning with IFN-γ also influences the therapeutic potential of EVs. Thus, a previous study found that stimulation of MSCs with IFN-γ alters the expression profile of miRNAs contained in the MSC-EVs. It should be noted that, among the miRNAs found to be significantly differentially expressed, many of them have been demonstrated to regulate cytokine production, T cell activation and other immunological processes (Zhao et al., 2017; Mas-Bargues and Borras, 2021). Furthermore, in a rat model of *E. coli*–induced pneumonia, EVs derived from MSCs primed with IFN-γ exhibited an enhancement of macrophage phagocytosis and killing of bacteria and an increased ability to restore capillary endothelial barrier function as compared to EVs from naive MSCs (Varkouhi et al., 2019; Table 4).

Preconditioning of MSCs with TNF-α has also demonstrated beneficial effects increasing the angiogenic activity *in vitro* and *in vivo* (Kwon et al., 2013) with increased expression of VEGF (Crisostomo et al., 2008) and bone morphogenetic protein-2 (BMP-2) (Lu et al., 2013). Although the effects induced by TNF-α are apparently less pronounced compared to those found with IFN-γ (Hemeda et al., 2010; Prasanna et al., 2010), the combination of both cytokines may have synergistic effects, significantly increasing the secretion of the complement inhibitor factor H (Tu et al., 2010) or the immunomodulatory molecules TGF-β and HGF (Ryan et al., 2007). Recently, it has been reported that the combination of IFN-γ and TNF-α is indeed more effective upregulating the expression of several immunosuppressive molecules [IDO, programmed death ligand-1 (PD-L1) and HLA-G] which could increase the immunosuppressive potential of MSCs (Ma et al., 2014; Jin et al., 2016) by promoting M2 macrophage differentiation (Francois et al., 2012) and Treg activation (Li et al., 2015b). In line with these evidences, EVs derived from MSCs treated with a combination of IFN-γ and TGF-β were found to promote more efficiently the transformation of mononuclear cells into T regulatory cells, through a mechanism mediated by IDO induction (Zhang et al., 2018; Mas-Bargues and Borras, 2021).

Other cytokines used tested for preconditioning of MSCs include IL-1β or IL-17. Pre-treatment of MSCs with IL-1β increases expression of multiple cell adhesion molecules, which in turn facilitate the migration of MSCs to the site of injury/inflammation, but also enhances the production of numerous chemokines which are capable of recruiting immune cells and could facilitate their infiltration in order to repair tissue injury (Carrero et al., 2012). Moreover, IL-1β-primed human MSCs also showed a higher ability to migrate to inflammatory sites through upregulation of CXC chemokine receptor type 4 (CXCR4) and a better therapeutic capacity due to an increased ability to induce M2 macrophage polarization and a switch from a Th1/Th17 to a Th2/Treg profile (Fan et al., 2012). In a recent study, it was demonstrated that IL-1β further increased the immunoregulatory activity by MSCs pretreated with a combination of TNF-α and IFN-γ (Hackel et al., 2021).

IL-17 is another pro-inflammatory cytokine used in some studies. However, despite its key role in the pathogenesis of several inflammatory diseases, the effects of IL-17 priming on MSCs is not completely understood. Thus, whereas some studies have shown that IL17-treated MSCs exert an enhanced ability to inhibit T-cell proliferation and the expression of Th1 cytokines (Sivanathan et al., 2015), other studies have shown the opposite effects (Tian et al., 2016). In contrast, another study showed that a combination of IFN-γ/TNF-α and IL-17 increased the ability of MSCs to reduce T-cell proliferation in an iNOS-dependent pathway (Han et al., 2014).

### Treatment of Mesenchymal Stem Cells With Toll-Like Receptor Ligands

Mesenchymal stem cells sense the environment through various damage-associated and pathogen-associated cell surface receptors and respond differentially depending on the environmental requirements (Waterman et al., 2010; Mastri et al., 2012; Rolandsson Enes et al., 2021). For example, stimulation of TLR3 and TLR4 appeared to polarize MSCs into two different immune regulatory phenotypes. Whereas treatment with the TLR4 agonist LPS induced a polarization of MSCs toward a more pro-inflammatory phenotype (with increased release of IL-6 and IL-8), priming with the TLR3 agonist Poly (I:C) activated a immunosuppressive phenotype, with increased expression of IDO, PGE2 and RANTES (Waterman et al., 2010; Fuenzalida et al., 2016; Rolandsson Enes et al., 2021).

Treatment with endotoxin has been shown to increase the release of proangiogenic factors (such as VEGF, FGF2, IGF-1 or HGF) in MSCs (Crisostomo et al., 2008; Grote et al., 2013). The activation of TLR4 has also been demonstrated to be critical for MSCs survival and therapeutic effect in a pre-clinical model of *E. coli*-induced ALI (Table 4). In this study, MSCs isolated from TLR4 deficient mice had impaired survival under conditions of inflammatory stress *in vitro* and were not therapeutically active *in vivo*. Mechanistically, it was shown that TLR4 pathway regulates signaling through PAR1 on MSCs and TLR4 stimulation leads to expression and secretion of prothrombin (Gupta et al., 2018). On the other hand, priming with the TLR-3 agonist Poly (I:C) was shown to increase the secretion of EVs with a higher content in KGF and to augment bacterial clearance in a murine model of *E. coli*-induced pneumonia (Monsel et al., 2015). When examined *in vitro*, EVs from Poly (I:C)-primed MSC were more effective in enhancing the anti-inflammatory and phagocytic activity of cultured macrophages, possibly by transferring COX-2 mRNA to activated monocytes, resulting in an increase in production of PGE2 (Monsel et al., 2015). Moreover, the preconditioning with the TLR-3 agonist MSC-EVs significantly reduced lung protein permeability, an effect which was attributed to its Ang-1 content, a protein with well-known anti-inflammatory, anti-permeability, and endothelial protective characteristics (Park et al., 2019).

### Genetic Engineering of Mesenchymal Stem Cells

Another approach used to improve the survival and homing of MSCs to the injured tissue and to enhance their therapeutic potential is genetic modification (Baldari et al., 2017; Damasceno et al., 2020). In the context of ARDS, special attention has been paid to increase the anti-inflammatory effects of MSCs and their ability to repair the alveolar-capillary barrier. Thus, gene modification has been used to increase the homing and immunomodulatory properties of MSCs (Table 4). For example, overexpression of CXCR4 significantly enhanced the homing and colonization of damaged lung tissue by MSCs which resulted in a decrease in the levels of inflammatory cytokines and the neutrophil count in a model of LPS-induced lung injury (Yang et al., 2015). Transduction of IL-10 also enhanced the beneficial effects induced by MSCs, as evidenced by the significant reduction of infiltrated neutrophils, the increase phagocytic capacity of AM and the reduction in markers of structural lung injury in a model of bacterial pneumonia (Jerkic et al., 2019) or acid-induced lung injury (Islam et al., 2019). Overexpression of soluble IL-1 receptor-like-1 (sST2), a decoy receptor for IL-33, also caused a substantial decrease in lung airspace inflammation and vascular leakage and preserved alveolar architecture (Martinez-Gonzalez et al., 2013).

Genetic engineering has also been used to increase the angiogenic potential of MSCs and facilitate the repair of the alveolar epithelium. As discussed in previous section, these effects are mediated by the secretion of factors such as HGF or Ang-1. In line with these evidences, overexpression of HGF increased MSCs viability and led to a significant improvement in oxygen saturation and lung edema in a rat model of ischemia/reperfusion-induced lung injury (Chen et al., 2017). Overexpression of HGF also increased the efficacy of MSCs in models of acid-(Islam et al., 2019) and radiation- (Wang et al., 2013) induced lung injury. Similarly, MSCs overexpressing Ang-1 demonstrated a further reduction of alveolar inflammation as compared to untreated MSCs (Mei et al., 2007). In another study, transfection of Ang-1 enhanced the anti-inflammatory effects and improved the endothelial barrier protective effects induced by MSCs in an *in vivo* endotoxin-induced lung injury (Xu et al., 2008). In animal models of ALI induced by endotoxin, overexpression of PGE receptor 2 (Han et al., 2016) or heme oxygenase-1 (Chen et al., 2019) also increased anti-inflammatory effects induced by MSCs and enhanced the recovery of the alveolar-capillary barrier reducing the formation of edema. Overexpression of p130 (a member of the retinoblastoma gene product family) or E2F4 (a transcriptional repressor in conjunction with p130) increased the retention of MSCs in the lungs of mice treated with endotoxin and promoted the differentiation of MSCs into ATII cells, facilitating the reduction pulmonary edema and lung fibrosis, thereby improving the protective effects of MSCs in this model of ARDS (Zhang X. et al., 2019).

Finally, enrichment of selective miRNAs has also been proven to increase the therapeutic potential of MSCs in ARDS models. For example, EVs derived from microRNA-30b-3p-overexpressing MSCs protected against LPS-induced ALI by improving histopathology, decreasing the alveolar barrier permeability, the neutrophil infiltration, lung MPO activity and reducing alveolar inflammation (Yi et al., 2019). EVs enriched in miR-22-3p also reduced lung inflammation and oxidative stress and inhibited epithelial and endothelial apoptosis in endotoxin-treated rats (Zheng et al., 2021).

## Discussion

**FIGURE 1:**
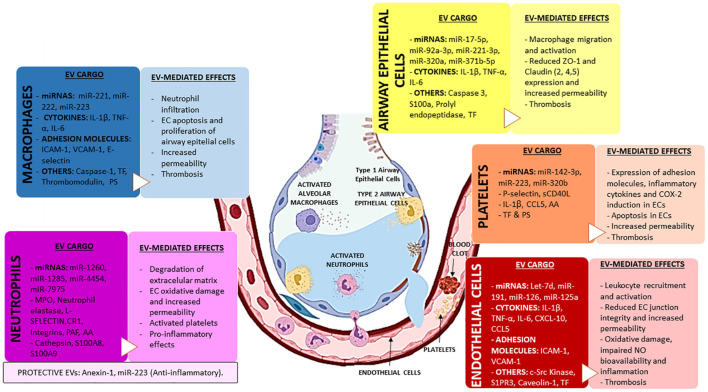
Extracellular vesicles (EVs) involved in the pathophysiology of acute respiratory distress syndrome (ARDS). Schematic representation of an injured alveolus in ARDS and the potential role of specific EVs based on their parent cell and their cargo. AA, Arachidonic acid; CCL5, C-C motif chemokine 5 (also known as RANTES); CR1, Complement Receptor-1; CXCL-10, C-X-C motif chemokine ligand 10; EC, endothelial cells; IL, interleukin; ICAM-1, intercellular adhesion molecule-1; miR, micro ribonucleic acid; NO, nitric oxide; PAF, platelet activating factor; PS, phosphatidylserine; S100A8, S100 calcium-binding protein A8; S100A9, S100 calcium-binding protein A9; S1PR3, Sphingosine-1-Phosphate Receptor 3; sCD40L, soluble CD40 ligand; TF, tissue factor; TNFα, tumor necrosis factor-α; VCAM-1, vascular cell adhesion protein 1; ZO-1, zonula occludens-1. This figure was created with BioRender.com.

Extracellular vesicles play a crucial role in the onset and progression of inflammation and pulmonary damage during ARDS. Extensive research has identified EVs released from structural (alveolar epithelial and endothelial cells) and immune cells with a potential pathophysiological role in the disease. Thus, EVs are increased following lung injury and have been shown to induce pulmonary inflammation by promoting the recruitment and activation of immune cells, to impair alveolar-capillary barrier function by altering intercellular junction and to contribute to the structural and functional damage by inducing apoptosis in AECs and endothelial cells (Figure 1 and Tables 2, 3). EVs derived from AECs, endothelial cells and platelets can also promote coagulation, leading to the formation of microthrombi in the lungs. Furthermore, the release of lung-derived EVs into the systemic circulation might contribute to damage in distal organs and DIC. However, although the field is rapidly progressing, standardized methods to isolate the different types of EVs and determine the cellular origin of EVs are still missing. These pitfalls lead to a lack of reproducibility between different studies and slow down our understanding of how changes in the release of specific EVs or the composition of their cargo translate into differences in the functional effects of these vesicles and their specific impact on the progression and resolution of ARDS.

**FIGURE 2:**
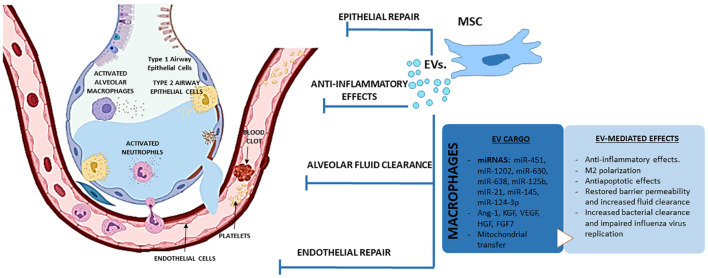
Therapeutic potential of extracellular vesicles (EVs) derived from mesenchymal stem cells (MSCs) in acute respiratory distress syndrome (ARDS). Schematic diagram showing the protecting effects of the protective effects induced by EVs derived from MSCs in ARDS. Potential mediators and described protective effects are shown in the right panels. Ang-1, angiopoetin-1; FGF7, fibroblast growth factor 7; HGF, hepatocyte growth factor; KGF, keratinocyte growth factor; M2, M2 or anti-inflammatory macrophages; miR, micro ribonucleic acid; MSC, mesenchymal stem cell; VEGF, vascular endothelial growth factor. This figure was created with BioRender.com.

Extracellular vesicles also offer a unique opportunity to develop new therapeutics for the treatment of ARDS. There is increasing attention in exploiting the therapeutic potential of EVs derived from MSCs. A large number of preclinical studies have demonstrated that these EVs are able to reduce pulmonary inflammation, stimulate alveolar fluid clearance and to attenuate the lung injury through mechanisms involving the transportation of regulatory molecules (VEGF, HGF, KGF, Ang-1 or specific miRNas) and the transfer of functional mitochondria (Figure 2 and Table 3). A summary of the main findings supporting the use of MSC-derived EVs for the treatment of ARDS is provided in Table 3. Preconditioning and genetic engineering strategies are currently being evaluated to improve the therapeutic potential of these EVs, as shown in Table 4. However, the heterogeneity among the MSCs and the EVs preparations (including differences in the source, intrinsic donors’ conditions or EVs isolation procedures), make comparisons among different studies difficult. Based on these promising preclinical studies, several clinical trials are currently evaluating the safety and efficacy of MSCs and/or MSC-EVs in patients with ARDS, including 5 clinical trials valuating the effects of MSC-EVs in patients with COVID-19^1^. First reports suggest that administration of MSCs is safe in patients with ARDS (Zheng et al., 2014) and may limit the cytokine storm (by reducing the levels of C-Reactive Protein, IL-6 or TNF-α) and improve oxygenation in severe COVID-19 patients (Sengupta et al., 2020). However, further studies are still needed to determine their optimal dosage, timing and route of administration.

In summary, EVs play an essential role in the pathophysiology of ARDS by modulating the onset and the progression of lung inflammation and injury. EVs also offer an extraordinary opportunity for developing novel therapeutic agents. EVs-based therapies, in contrast to administration of living cells, may be safer and easier to manage. Furthermore, MSC-EVs can be modified to deliver specific proteins or miRNA (Table 4) which could be directed to particular cell types by specific EV surface markers. Although this is an exciting field with a high translational potential, the implementation of standardized methods for EVs isolation and manipulation is essential in order to achieve reproducible results which may be validated in large multicenter clinical trials.

## Author Contributions

SE-R, LM, and RH conceived and designed the revision. SE-R, PG-R, LM, and RH contributed to writing of the manuscript. LM, RH, JAL, and FP-V revised the manuscript and approved the final version of the manuscript. All authors contributed to the article and approved the submitted version.

## Conflict of Interest

The authors declare that the research was conducted in the absence of any commercial or financial relationships that could be construed as a potential conflict of interest.

## Publisher’s Note

All claims expressed in this article are solely those of the authors and do not necessarily represent those of their affiliated organizations, or those of the publisher, the editors and the reviewers. Any product that may be evaluated in this article, or claim that may be made by its manufacturer, is not guaranteed or endorsed by the publisher.

## Footnotes

1
https://clinicaltrials.gov/

## Glossary

2MG: α 2-macroglobulin
ACE-2: angiotensin-converting enzyme 2
ADAM: a disintegrin and metalloproteinase domain containing proteins
ADAMTS: ADAM with thrombospondin domains
AECs: alveolar epithelial cells
AJ: adherens junctions
Akt: protein kinase B
ALI: acute lung injury
AM: alveolar macrophages
Ang-1: angiopoietin 1
ARDS: acute respiratory distress syndrome
ATI/II: alveolar type I/II
ATP: adenosine triphosphate
BALF: bronchoalveolar lavage fluid
Bcl-2: B-cell lymphoma 2
BMP-2: bone morphogenetic protein-2
CCL: chemokine (C-C motif) ligand
CCN1: cellular communication network factor 1
CD: cluster of differentiation
CLDN: claudin
CM: conditioned medium
COX: cyclooxygenase
CR: complement receptor
CXCL: chemokine (C-X-C motif) ligand
CXCR: CXC chemokine receptor
c-Src: cellular Src
DIC: disseminated intravascular coagulation
DNA: deoxyribonucleic acid
ECM: extracellular matrix
EGFR: epidermal growth factor receptor
eNOS: endothelial nitric oxide synthase
EPO: erythropoietin
ERK1/2: extracellular signal-regulated kinase ½
ESCRT: endosomal sorting complexes required for transport
EVs: extracellular vesicles
F-actin: filamentous actin
FGF: fibroblast growth factor
fMLP: formyl-methionine-leucine-phenylalanine
Gal-9: galectin-9
HGF: hepatocyte growth factor
HLA: human leukocyte antigen
HLMVEC: human lung microvascular endothelial cell
HO-1: heme oxygenase-1
HPMEC: human pulmonary microvascular endothelial cell
ICAM-1: intercellular adhesion molecule 1
ICU: intensive care unit
IDO: indolamine 2,3 deoxygenase
IFN- γ: interferon gamma
IGF: insulin-like growth factor
IL: interleukin
IM: interstitial macrophages
I/R: ischemia-reperfusion
ISEV: international society of extracellular vesicles
JNK: c-JunN-terminal kinase
KFG: keratinocyte growth factor
LFA-1: lymphocyte function-associated antigen 1
LPS: lipopolysaccharide
Mac-1: macrophage-1 antigen or macrophage integrin
MCP: monocyte chemotactic protein
MerTK: Mer tyrosine kinase
MIP: macrophage inflammatory protein
MLC: myosin light chain
MMP: matrix metalloprotease
MPO: myeloperoxidase
MSCs: mesenchymal stem/stromal cells
Myd88: myeloid differentiation primary response 88
NETs: neutrophil extracellular traps
NF κ B: nuclear factor kappa B
NLRP3: nod-like receptor family pyrin domain containing 3
NO: nitric oxide
PAR-1: protease-activated receptor-1
PD-L1: programmed death ligand-1
PECAM: platelet endothelial cell adhesion molecule
PGE: prostaglandin E
PI3K: phosphatidylinositol 3-kinase
PS: phosphatidylserine
RANTES, regulated on activation: normal T cell expressed and secreted
RNA: ribonucleic acid
ROCK: rho-associated protein kinase
ROS: reactive oxygen species
S1PR: sphingosine-1-phosphate receptor
SARS-CoV2: severe acute respiratory syndrome coronavirus 2
SDF: stromal cell-derived factor
SOCS: suppressor of cytokine signaling
sPLA2: secretory phospholipase A2
sST2: soluble IL-1 receptor-like-1
STAT: signal transducer and activator of transcription
TF: tissue factor
TFPI: tissue factor pathway inhibitor
TGF- β: transforming growth factor β
Tie-2/TEK: angiopoietin receptor
TIMP: tissue inhibitor of metalloproteinases
TJs: tight junctions
TLR: toll-like receptor
TNF- α: tumor necrosis factor α
Tregs: regulatory T cells
tRNA: transfer RNA
TSG101: tumor susceptibility gene 101
VCAM-1: vascular cell adhesion molecule 1
VE-cadherin: vascular endothelial-cadherin
VEGF: vascular endothelial growth factor
VILI: ventilator-induced lung injury
ZO-1: zonula occludens 1.
